# Ventricular Arrhythmias in the Patient with a Structurally Normal Heart

**DOI:** 10.19102/icrm.2018.091004

**Published:** 2018-10-15

**Authors:** Daniel A. Sohinki, Sunil T. Mathew

**Affiliations:** ^1^Department of Cardiology, University of Oklahoma Health Sciences Center, Oklahoma City, OK, USA; ^2^Heart Rhythm Institute, University of Oklahoma Health Sciences Center, Oklahoma City, OK, USA

**Keywords:** Premature ventricular contraction, structural heart disease, structurally normal heart, ventricular arrhythmia

## Abstract

Ventricular arrhythmias (VAs) are among the most common cardiac rhythm disturbances encountered in clinical practice. Patients presenting with frequent ventricular ectopy or sustained ventricular tachycardia represent a challenging and worrisome clinical scenario for many practitioners because of concerning symptoms, frequent associated acute hemodynamic compromise, and the adverse prognostic implications inherent to these cases. While an underlying structural or functional cardiac abnormality, metabolic derangement, or medication toxicity is often readily apparent, many patients have no obvious underlying condition, despite a comprehensive diagnostic evaluation. Such patients are diagnosed as having an idiopathic VA, which is a label with specific implications regarding arrhythmia origin, prognosis, and potential for pharmacologic and invasive management. Further, a subset of patients with otherwise benign idiopathic ventricular ectopy can present with polymorphic ventricular tachycardia and ventricular fibrillation, adding a layer of complexity to a clinical syndrome previously felt to have a benign clinical course. Thus, this review seeks to highlight the most common types of idiopathic VAs with a focus on their prognostic implications, underlying electrophysiologic mechanisms, unique electrocardiographic signatures, and considerations for invasive electrophysiologic study and catheter ablation. We further address some of the data regarding idiopathic ventricular fibrillation with respect to the heterogeneous nature of this diagnosis.

## Introduction

Idiopathic ventricular arrhythmias (VAs) are rhythm disturbances arising in patients without structural heart disease (SHD).^1^ Presentations range from intermittent premature ventricular complexes (PVCs) to sustained ventricular tachycardia (VT). PVCs are the most common nonsustained cardiac rhythm disturbance in adults, with an estimated prevalence of 40% to 75% on 24- to 48-hour Holter monitoring.^2^ Sustained VT in patients without SHD is less common, accounting for 10% of all patients referred for the evaluation of VT.^1,3^ VAs can occur in a wide variety of clinical settings [eg, in patients with cardiomyopathy, coronary artery disease (CAD), left ventricular hypertrophy (LVH), metabolic derangements, medication toxicity, and conditions associated with elevated sympathetic tone^2,4,5^]. Studies consistently point to frequent VAs as an adverse prognostic marker in these conditions.^6–10^ However, data in patients without SHD are inconsistent, creating controversy regarding optimal evaluation and management.^11^ Further, a subset of patients without SHD present with polymorphic VT and ventricular fibrillation (VF), and no current risk-prediction strategy exists to accurately identify high-risk patients. Accordingly, a detailed understanding of VAs arising in patients without SHD is important in order to assess prognosis and identify those who would benefit from the suppression of ventricular ectopy. Thus, the goal of this review article is to summarize the available evidence on the genesis and clinical characteristics of VAs in patients without SHD. It should be noted that, while patients with malignant VAs due to inherited arrhythmia syndromes lack SHD, such conditions are outside the scope of this review.

## General prognostic implications

The prognostic significance of VAs in patients without SHD remains unclear. Several studies involving apparently healthy subjects conducted in the 1970s and 1980s reported no significant increases in mortality during long-term follow-up of patients with PVCs, though the absence of SHD was documented on clinical grounds and cardiac imaging was not routinely performed.^12–14^ In contrast, a study of 15,637 white males reported a threefold increase in the risk of sudden cardiac death (SCD) in participants with PVCs, with frequent and “complex” (ie, multifocal nonsustained VT, or R-on-T) PVCs predicting a higher risk of SCD.^15^ While patients in the study with any PVCs were largely without clinically apparent CAD (only three CAD-related deaths occurred in this group), a formal evaluation for underlying SHD was not documented. Similarly, the analysis of data from the Framingham cohort as well as of a large cohort of apparently healthy Japanese adults found a twofold increased risk of all-cause mortality among males with frequent and complex PVCs, though also without a formal assessment for SHD.^16,17^ Interestingly, neither cohort demonstrated an adverse effect on prognosis in females.^16,17^ A large meta-analysis published in 2012 found increased odds of all-cause and cardiovascular mortality, SCD, and ischemic heart disease in patients with PVCs (odds ratio: 1.72).^11^ However, the majority of studies in the analysis excluded heart disease on clinical and electrocardiographic grounds and only one of the 10 included studies involved an echocardiographic assessment.^11^ Additionally, regression analysis showed that age and other traditional Framingham risk factors were correlated with adverse outcomes among participants with PVCs, suggesting that the association may have been related with undiagnosed SHD.^11^

## Outflow tract tachycardias

### Embryologic considerations

Right ventricular outflow tract (OT) (RVOT) and left ventricular OT (LVOT) VAs are the most common subtypes of idiopathic VA.^18^ While patients with these conditions have no apparent risk of SHD, an understanding of the embryologic development of the OT can provide clues as to the origin of these arrhythmias. OT formation occurs during looping of the heart from pharyngeal mesodermal progenitor cells.^19–21^ As the heart develops, a significant lengthening of the RVOT occurs as compared with the LVOT, mainly in the area of the subpulmonary myocardium.^18^ After looping of the heart is complete, several primitive areas of cardiac conduction tissue can be identified histologically, with a significant amount localized in the area of the developing OTs.^18^ While the conduction properties of these tissues in human embryogenesis remain poorly understood, animal models have demonstrated relatively slow conduction through these areas.^22^ Animal models have also demonstrated localized variations in the expression of certain ion channels and reporter genes (eg, CCS-*lacZ*^18,23^ and MinK-*lacZ*^24^) that are responsible for maturation of the conduction system. It is hypothesized that the predilection of these sites for the development of VAs may result from regional variations in embryologic gene expression, reexpression of these genes in adulthood, enhanced activity of remaining embryonic primitive conduction tissue, or a combination of these mechanisms.^18^ Histologic studies have also noted a preponderance of midmyocardial M cells in the RVOT, which are believed to be more susceptible to delayed afterdepolarizations (DADs) and thus may act as triggers for episodes of tachycardia originating in this area.^25^

### Cellular mechanisms—adenosine sensitivity

Early series of patients with adenosine-sensitive VT were reported in the 1980s and 1990s.^26,27^ Because adenosine has little direct effect on ventricular myocardium,^28^ it was postulated that it exerted its antiarrhythmic effect by blocking catecholamine-mediated cyclic adenosine monophosphate (cAMP) production in ventricular myocytes. Experimental studies have confirmed the ability of adenosine to suppress DADs induced by forskolin (an adenylate cyclase activator) but not those induced by dibutyryl cAMP, indicating that the site of adenosine’s antiadrenergic effect is at the level of cAMP production.^29^ These effects are thought to be mediated through beta-receptor-coupled inhibitory G-proteins, and subsequent studies have confirmed the ability of inactivators of inhibitory G-proteins (eg, pertussis toxin) to blunt adenosine’s antiarrhythmic effect.^29^ Finally, the ability of adenosine to inhibit DADs is specific to cAMP-mediated calcium handling, as triggered activity resulting from the inhibition of sodium−potassium adenosine triphosphatase (eg, by ouabain) is insensitive to adenosine administration.^30^ Thus, adenosine is felt to exert its antiarrhythmic effects through the activation of the A_1_ receptor, subsequent activation of beta-receptor-coupled inhibitory G-proteins, and the antagonism of adenylate–cyclase-mediated cAMP production.^30,31^

The downstream effects of adenosine are antagonism of beta-adrenergic stimulation of L-type calcium channels by protein-kinase A (PKA) as well as calcium-induced calcium release from the sarcoplasmic reticulum (SR).^32^ Through its reduction in PKA activity, adenosine reduces phosphorylation of the ryanodine receptor and phospholamban (the cell’s inhibitor of sarcoplasmic reticulum calcium adenosine triphosphatase) actions, which reduce the amount of cytosolic calcium that is available for excitation–contraction coupling.^31^ Further, beta stimulation enhances activity of the delayed rectifier potassium current (*I*_Ks_) and transient inward current (*I*_Ti_), both of which are antagonized by adenosine.^33^ In the series published by Wilber et al.,^27^ endocardial mapping identified sites of early activation in the RVOT of all involved patients, providing early evidence of involvement of the OTs in these arrhythmias.

Since these early studies, the importance of triggered activity and DADs has been recognized as fundamental to the initiation of OT tachycardias.^31^ Notably, a variety of arrhythmia syndromes can result from triggered activity related with DADs, though the mechanism underlying intracellular calcium overload differs significantly. The designation of OT tachycardias as “adenosine-sensitive” underscores their critical dependence on increased intracellular cAMP resulting from beta-adrenergic stimulation.^31^ As alluded to above, embryologic differences between OTs and the rest of the myocardium may explain the susceptibility of myocardial cells at these sites to triggered activity, though other factors are likely important as well. Transcriptional profiling has demonstrated regional differences in gene expression related to intracellular calcium handling and calcium channel production. Hasdemir et al. noted downregulation of calcium-regulatory genes in the septal wall of the RVOT in comparison with in other sites, providing evidence that regional differences in the regulation of intracellular calcium handling are likely under genetic, metabolic, and hormonal control.^33^

### Clinical characteristics

The majority of OT tachycardias originate from the RVOT, though 10% to 20% originate from the LVOT.^18,30^ OT tachycardias typically present as (1) paroxysmal or sustained monomorphic VT; (2) frequent episodes of nonsustained VT; or (3) frequent, isolated PVCs.^1,34^ The clinical presentation can vary widely, from asymptomatic episodes detected on 12-lead electrocardiogram (ECG) or ambulatory monitoring to palpitations and presyncope.^18,35^ Frank syncope is felt to be an uncommon presentation, occurring in less than 10% of cases.^35^ Patients typically present in the third to fifth decade of life and are more commonly female, with sex-specific differences in situations that exacerbate ventricular ectopy.^35,36^ Women with OT tachycardias typically report a worsening of symptoms at times of hormonal flux, including the premenstrual, peripartum, and perimenopausal periods, and during the administration of oral contraceptives. In contrast, men typically report symptoms during periods of exertion or emotional stress.^36^

While OT tachycardias are generally considered to be “benign” arrhythmias, there have been several reports of patients with otherwise typically isolated OT ectopy who presented with spontaneous VF or polymorphic VT.^25^ Though it was previously felt that the PVC coupling interval alone could be used to distinguish the benign from malignant form of OT tachycardia, a relatively large case series demonstrated that neither the coupling interval nor the burden of ventricular ectopy could identify patients with RVOT ectopy at risk for malignant VAs.^37^ As noted above, although DADs are a common trigger for VAs in a variety of clinical syndromes, the presenting arrhythmia can vary (eg, bidirectional VT in catecholaminergic polymorphic VT and digitalis toxicity, PVCs and monomorphic VT in OT tachycardia, and a transition to polymorphic VT being common to all syndromes).^25^ Recent experimental studies^25^ suggest that an endocardial versus epicardial location of the ectopic focus has a significant impact on the presenting arrhythmia. Epicardial ectopic beats cause a reversal of the direction of transmural myocardial activation. This leads to a dramatic increase in transmural dispersion of refractoriness, as epicardial myocytes (cells with the shortest action potential duration) are now activated first instead of last. This creates a large vulnerable window, which allows subsequent closely coupled ectopic beats to initiate polymorphic VT.^25^ In the case series published by Noda et al., patients with otherwise benign RVOT ectopy who experienced polymorphic VT frequently had a first ectopic beat with a relatively long coupling interval, followed by very closely coupled second and third ectopic beats and salvos of polymorphic VT.^25,37^ Thus, a patient with OT tachycardia may present with a benign or malignant VA, depending on the origin of the ectopic focus and its effect on ventricular repolarization.^37,38^ Finally, frequent OT ectopy can be a precipitant of tachycardia-mediated left ventricular (LV) dysfunction and treatment of ectopy in these cases can be curative.^39,40^

### Anatomic considerations and electrocardiogram characteristics

**Figure 1:**
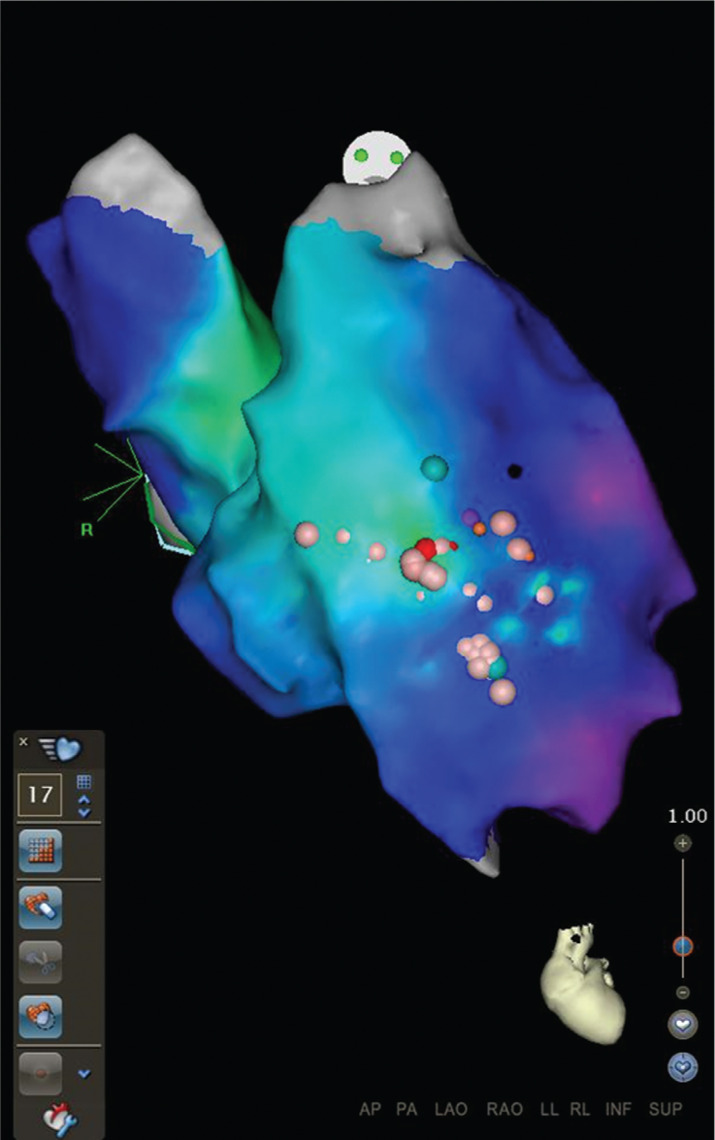
Fast anatomical mapping map of the RVOT and LVOT in a patient with RVOT VT (anteroposterior projection). Note the relative leftward and anterior position of the RVOT as compared with the LVOT. Created using CARTO^®^ 3 (Biosense Webster, Diamond Bar, CA, USA).

An understanding of the anatomical relationship between the RVOT and LVOT is crucial for determining the site of origin of an OT tachycardia based on the surface ECG. In the structurally normal heart, the RVOT courses anteriorly to the LVOT from a rightward-inferior to leftward-superior direction such that the superior portion of the RVOT is leftward of the LVOT **(Figure 1)**.^18^ As noted above, because the RVOT lengthens to a greater degree than the LVOT during embryonic development and because of the effect of muscular subpulmonary infundibulum, the pulmonary valve is located more superiorly than the aortic valve.^18^ Notably, the trabeculum septomarginalis (TSM) represents the most caudal contribution of the bulbus cordis to the RVOT, making any portion of the RVOT located rostral to this area (eg, crista supraventricularis; anterior, septal, and lateral RVOT) a possible site of origin for RVOT tachycardia.^18,41^ Importantly, while many authors reference a “septal” surface of the RVOT, only the very proximal portion of the RVOT near the TSM contributes to the interventricular septum. Distally, the portion of the RVOT that faces septally is not in continuity with the septal myocardium or LVOT.^42^ Additionally, myocardial “sleeves” are commonly seen to extend above the pulmonary valve into the proximal main pulmonary artery, making these possible sites of VA origin as well.^43^ In one autopsy series, more than 50% of RVOT specimens had supravalvular extensions, with a relatively symmetric distribution between the three cusps.^43^ While short sleeves of myocardium also extend above the aortic valve, their distribution is more asymmetric as compared with in the case of the RVOT, with most of the myocardial extensions noted at the base of the right coronary cusp (RCC) and left coronary cusp (LCC) [< 1% above the noncoronary cusp (NCC)].^43,44^ Tachycardia originating above the pulmonary valve is relatively rare, with the majority of RVOT tachycardias stemming instead from the anterior and superior septal aspects of the RVOT, just inferior to the pulmonary valve.^45^ Similarly, LVOT tachycardias can originate from any site above or below the aortic valve, with an estimated 9% to 24% of idiopathic VT originating from the sinuses of Valsalva.^46^

**Figure 2:**
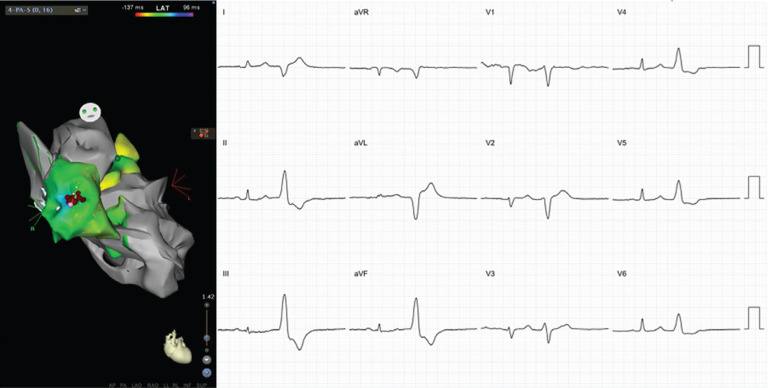
ECG and anatomic location of RVOT PVCs. The left pane shows an anteroposterior projection of the RVOT, LVOT, and LV geometry. Note the precordial transition after V3. The PVC is QS in lead I, reflecting the proximal location in the RVOT as noted in the CARTO^®^ image, resulting in net left-to-right activation. These images were created using CARTO^®^ 3 and the CARTOSound™ enhanced mapping feature (Biosense Webster, Diamond Bar, CA, USA).

As noted above, OT tachycardias generally present with a left bundle branch morphology (ie, QS or rS in lead V1) and an inferior axis (ie, predominant positive forces in leads II, III, and aVF), with several additional ECG features being helpful in pinpointing the site of origin.^46^ In general, a later precordial transition increases the likelihood that the arrhythmia is originating from the RVOT,^46^ which is a reflection of the standard positioning of the precordial leads starting over the right anterior chest and coursing posterolaterally **(Figure 2)**. Because lead V1 is positioned in the right parasternal area (and thus directly over the anterior RVOT), VTs originating from this area will produce net forces posterolaterally. This production results in the predominant negative deflections seen in the early precordial leads. As the site of origin moves posteriorly in the OTs, early ventricular activation is expected to produce a relatively greater degree of anteriorly directed forces, resulting in greater and earlier positive deflections in the early precordial leads. A commonly referenced criterion for RVOT origin is a transition to an R/S ratio of greater than 1 after V3, though this rule is not absolute. In one study, 95% of arrhythmias with a free-wall origin in the RVOT demonstrated a delayed precordial transition versus just 21% of septal RVOT VTs.^47^ This finding possibly reflects the more posterior location of the septal portion of the superior RVOT. Tachycardias originating from the septal surface of the RVOT are noted to be taller and narrower (< 140 ms) than anterior or free wall VTs and are also less likely to demonstrate QRS notching.^42,47^ Finally, an R-wave in lead I is more indicative of a septal and posterior origin as compared with an anteromedial or free wall origin.^42,47^ Tachycardias originating above the pulmonary valve tend to have very strong inferior axes, with R in lead III being taller than in lead II and S being more negative in aVL than in aVR.^42^

**Figure 3:**
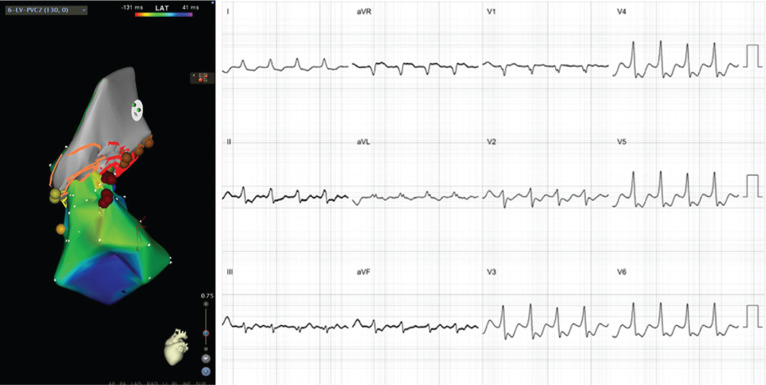
ECG and anatomic location of LVOT VT. The left pane shows a left lateral projection of the LVOT beneath the LCC and NCC. Note the earlier precordial transition as compared with RVOT ectopy. These images were created using CARTO^®^ 3 and the CARTOSound™ enhanced mapping feature (Biosense Webster, Diamond Bar, CA, USA).

**Figure 4:**
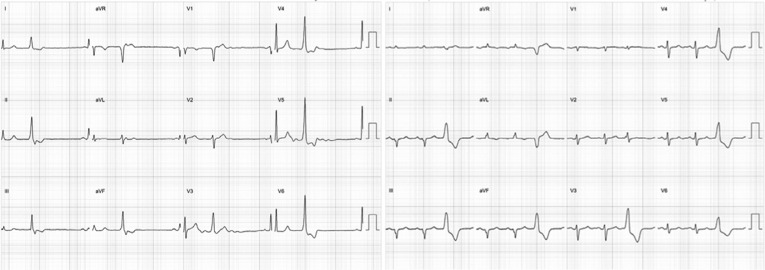
RCC PVC (left) and LCC PVC (right). Note the narrower QRS in the RCC PVC with relatively less notching as well as the relatively later precordial transition as compared with the LCC PVC. Also note the R-wave in lead I.

Tachycardias originating from the LVOT exhibit an earlier precordial transition as compared with the RVOT **(Figure 3)**, though VTs originating from the superior septal RVOT can be difficult to distinguish from aortic cusp VTs because of their close anatomic proximity.^42^ While QRS duration is not useful in distinguishing one from the other, RVOT VT has a narrower R-wave and a smaller R/S ratio in leads V1 and V2 as compared with cusp VT.^48,49^ As noted above, a precordial transition before or after lead V3 can help to distinguish LVOT from RVOT tachycardia, though a transition at lead V3 presents a challenge and occurs in as many as 58% of OT tachycardias.^42^ Yamauchi et al. reported a V2 transition ratio [R/(R+S)_VT_ / R/(R+S)_NSR_] of less than 0.6, and precordial transition during arrhythmia later than the transition during sinus rhythm suggests RVOT origin.^42,45^ Other observations can be useful for localizing the exact site of origin within the LVOT as well. Tachycardias originating from LCC exhibit a greater degree of notching than do those originating from the RCC, and can present with either an “M” or “W” pattern in lead V1.^47,48^ An earlier precordial transition (leads V1 or V2) is suggestive of an LCC origin, whereas a V3 transition suggests an RCC origin.^50^ LCC origin is also indicated by a QS or rS in lead I, whereas RCC origin more often presents with positive forces in this lead **(Figure 4)**.^47^ Finally, one study demonstrated that a QR pattern in lead V1 is suggestive of an origin in the aortomitral continuity (AMC), as the left fibrous trigone prevents initial lateral wall activation, though tachycardia from this region should present with right bundle branch block (RBBB) morphology.^51^

### Electrophysiology study and ablation

The performance of invasive electrophysiology study in patients with OT tachycardia may be challenging, with suppression of the arrhythmia frequently seen due to sedation/anesthesia, recent use of antiarrhythmic drugs, and the artificial environment of the laboratory.^42^ Consequently, such studies are ideally performed with the patient awake, with antiarrhythmic drugs held periprocedurally if tolerated to allow for metabolic clearance. Programmed stimulation is then used with or without support from beta-agonists like isoproterenol. Commonly used protocols involve incremental burst pacing from the right ventricular apex or RVOT and/or single, double, or triple ventricular extrastimuli delivered at varying cycle lengths.^42^ Programmed stimulation is less successful than reentrant VT, resulting in VT initiation in approximately 65% of cases.^42^ Patients presenting with sustained monomorphic VT are more likely to have inducible VAs (approximately 75% of cases) as compared with patients with only single PVCs (approximately 4%).^52^ In noninducible cases, atropine and aminophylline may be administered to counteract the inhibitory effect of endogenous acetylcholine and adenosine on cAMP production.^42^ Ultimately, studies may be aborted if VAs are noninducible, with repeat procedures done at a later date.

### Activation mapping

**Figure 5:**
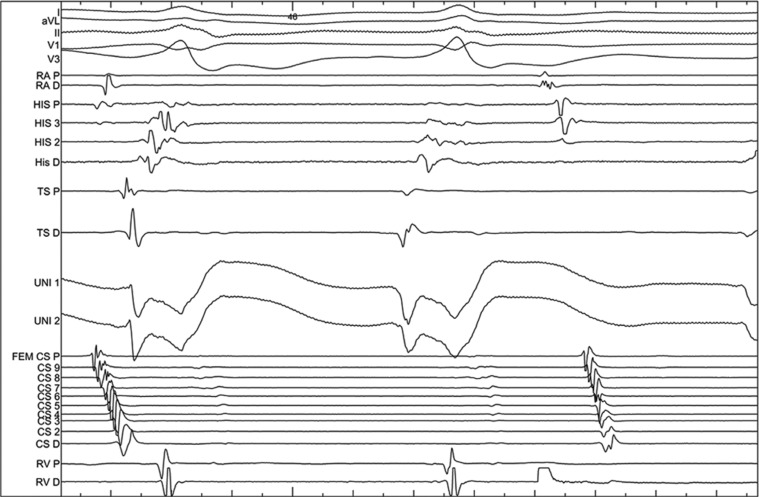
Intracardiac EGMs recorded during LVOT PVC mapping. Note the QS on the distal bipolar EGM of the mapping catheter as well as on both unipolar EGMs. Local ventricular activation precedes QRS onset by 50 ms at this site.

The initial preferred approach for mapping OT tachycardias depends on the frequency of VAs during the study and the degree of hemodynamic stability during the arrhythmia.^18^ If the patient has frequent VT or PVCs or is easily inducible, activation mapping is the preferred method.^18,42,53^ Whether using a point-by-point or multi-electrode catheter system, the general approach is to identify the earliest ventricular activation in relation to QRS onset.^42^ Local bipolar electrograms (EGMs) should precede QRS onset by at least 10 ms and be as long as 45 ms to 60 ms.^18,42^ In contrast to scar-related VAs, complex fractionated EGMs and mesodiastolic potentials are not a characteristic of OT tachycardias and suggest underlying SHD.^42^ Earliest activation is commonly indicated by a sharp QS signal, recorded on the unipolar EGM from the tip of the mapping catheter.^42^ However, this finding is sensitive, but not specific, for earliest activation.^42^ Approximately 70% of unsuccessful ablation sites in the RVOT manifest as a unipolar QS complex, though these sites frequently do not precede QRS onset.^42^ Ideally, the timing of onset of the bipolar- and unipolar-tip EGMs should be the same, indicating that the tip electrode is responsible for the initial negative portion of the bipolar EGM **(Figure 5)**.^42^ If mapping is performed above the semilunar valves, high-frequency, low-voltage recordings are often seen prior to local ventricular activation, which is thought to represent activation of the supravalvular myocardial extensions prior to ventricular activation.^18,42^ Electroanatomic mapping is a useful adjunct to conventional activation mapping because it provides a visual representation of the endocardial activation sequence relative to a reference signal and can help the electrophysiologist to visualize the site of earliest activation based on a color-coded map.^42^

### Pacemapping

Pacemapping involves the matching of the morphology of a paced QRS complex to the morphology seen during spontaneous arrhythmia. If pacing from a particular site results in a QRS complex that matches the spontaneous arrhythmia, then that site is presumed to be the site of origin.^18,42^ While ideally used to confirm the results of activation mapping, this approach can be used by itself if VAs are rare or difficult to induce.^42^ Spontaneous and paced QRS complexes should be compared using the same filtering settings as well as gain and sweep speed.^42^ A simultaneous side-by-side display should also be used to compare all leads.^42^ While subjective, semiquantitative comparisons are a useful first step, many mapping systems come with computerized software designed to compare the spontaneous and paced signals mathematically (eg, using correlation coefficients, root-mean-square error, or mean absolute deviation) to determine a “match percentage” between the two signals.^18,42^ Care should be taken to pacemap near the coupling interval or cycle length of the spontaneous VAs, as rate-dependent changes in QRS morphology may render pacemapping inaccurate.^18,42^ Further, when pacemapping above the semilunar valves, relatively higher pacing output is often required to capture myocardial sleeves in the proximal great vessels. This may result in the capture of relatively distant tissue, which can obscure arrhythmia origin (eg, capture of posterior RVOT tissue when pacing from the RCC).^42^

### Ablation

Once the site of earliest activation is identified, the application of radiofrequency (RF) current at 30 W to 40 W for 30 seconds to one minute is usually sufficient to destroy the targeted tissue (target tip–electrode temperature is 45°C–55°C when using nonirrigated catheters).^18,42^ Extensive tissue injury is usually not necessary, given the nondiseased nature of the target myocardium.^42^ When ablating in the aortic cusps, ablation is typically begun at a lower power (eg, 15 W) and gradually increased to achieve a tissue temperature of 50°C.^42^ Because of the risk of coronary injury, many operators will identify the ostia of the coronary arteries either via intracardiac echocardiography (ICE) or conventional aortography.^42^ Damage to the His bundle may also occur because of its proximity to the RCC and NCC, and a His-bundle EGM can frequently be recorded from these sites.^42^ Consequently, atrioventricular-nodal conduction should be monitored when ablating in these areas.

RF application typically results in a rapid ventricular rate with an identical morphology to that of the clinical tachycardia, followed by slowing and termination.^18,42^ While the response can be variable, arrhythmia termination is expected within 10 seconds.^18,42^ Ablation is acutely successful in greater than 90% of cases.^18,42^ A recurrence rate of approximately 7% to 10% has been found.^42^ The majority of recurrence occurs in the first 24 hours after ablation. Predictors of recurrence include poor pacemap match, exclusive reliance on pacemapping, and termination of VA inducibility by catheter trauma.^42^ As referenced above, catheter trauma may also cause nonclinically significant arrhythmias during the study, which can result in confusion during activation mapping or lead to hemodynamic instability. Recent studies suggest that remote magnetic catheter navigation may offer an advantage with respect to catheter-induced ectopy and reduce radiation exposure in the operator and patient.^49^ However, this technology is not available in many centers at this time.

## Fascicular tachycardias

### Mechanism and anatomic considerations

Zipes et al. first described fascicular VT in a case series in 1979, in which they reported on three patients without SHD who presented with VT with a RBBB morphology and left axis deviation that was inducible by rapid atrial pacing.^54^ Soon after, Belhassan et al. reported on the sensitivity of these VTs to verapamil (hence their alternative names of “verapamil-sensitive VT” and “Belhassan’s VT”).^55^ While the underlying mechanism was initially felt to be triggered activity, subsequent studies have demonstrated the arrhythmia to be reentrant in nature.^55,56^ This reentry is supported by the arrhythmia’s inducibility and termination through programmed stimulation and the ability to be entrained and reset with fusion.^57^

Evidence suggests that the underlying anatomic circuit involves macroreentry incorporating abnormal Purkinje tissue, with decremental conduction properties as the antegrade limb and the left posterior fascicle (LPF) as the retrograde limb.^57^ Maruyama et al. reported sequential activation throughout the cardiac cycle from the LV midseptum to the posteroapical septum in a patient with fascicular VT, with successful entrainment at multiple sites along the distal septum.^56^ These researchers’ findings lend further weight to the hypothesis that fascicular VT is a reentrant arrhythmia. Several studies have reported on a false tendon in the LV in the context of being part of the anatomic substrate for fascicular VT.^58,59^ Additionally, fascicular VT has been reported to be successfully ablated from a false tendon.^59^ The exact mechanism whereby a LV false tendon may give rise to VT is unclear, though studies have demonstrated them to have abundant histologically abnormal Purkinje tissue.^55^ It is hypothesized that the slow conduction necessary to sustain reentry results either from this tissue itself or from stretch exerted by the false tendon on normal Purkinje tissue in the LV septum.^60^ However, the relatively high prevalence of LV false tendons in the general population makes their specificity for fascicular VT as an underlying arrhythmia mechanism low.^61^

### Cellular mechanisms—verapamil sensitivity

While it has long been clinically observed that fascicular VT can be terminated by verapamil, little is known about the exact cellular mechanism. Electrical activation through Purkinje fibers is calcium-dependent, and the termination of VT by verapamil highlights the critical dependence of this arrhythmia on abnormal Purkinje tissue.^55^ In a canine model, verapamil reduced the activation potential amplitude and duration, and increased the tissue refractory period in Purkinje fibers.^62^ Further, verapamil is able to lessen the upstroke velocity of Purkinje fiber action potentials, increase the slope of phase II repolarization, diminish spontaneous Purkinje fiber activity, and lower the voltage overshoot during repolarization.^63,64^ These actions may be expected to exert an antiarrhythmic effect on Purkinje-dependent tachycardia. It is felt that diseased Purkinje in the proximal LV septum is the “verapamil-sensitive zone” that is susceptible to the antiarrhythmic effects of calcium-channel blockade.^57^

### Clinical characteristics

Fascicular VT represents 10% to 15% of all idiopathic VTs.^57^ Age at presentation is slightly younger than that seen with OT tachycardia at 15 years to 40 years, and males are more commonly affected than females.^57,65^ Typical presenting symptoms include paroxysms of palpitations, dizziness, and presyncope, with syncope being a less common presentation.^55^ Exacerbating symptoms are less well-defined as compared with those of OT tachycardia, with patients reporting symptoms more commonly at rest.^55^ However, presentations related to exertion and emotional stress have been reported as well.^55^ Similar to in the case of other tachyarrhythmia syndromes, tachycardia-mediated cardiomyopathy has been described in patients with incessant fascicular VT, and catheter ablation can be curative.^66^ While fascicular VT occurs in patients with structurally normal hearts, subclinical myocardial dysfunction can cause arrhythmias that may mimic fascicular VT. One series showed that, while a majority of patients with presumed fascicular VT had normal findings upon LV biopsy, a minority showed lymphocyte infiltration and fibrosis consistent with subclinical myocarditis.^67^ This raises the possibility that some patients diagnosed as having “idiopathic” fascicular VT may, in fact, have subclinical myocardial dysfunction that gives rise to their arrhythmia.

### Electrocardiogram features

**Figure 6:**
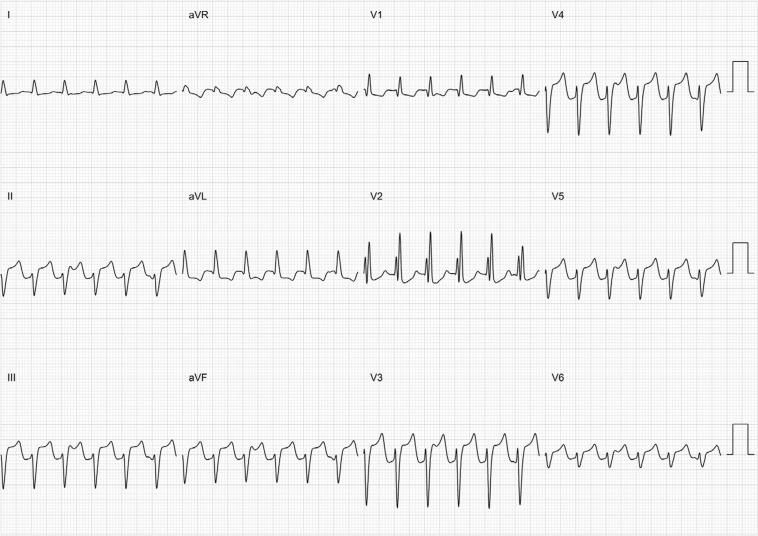
A typical example of fascicular VT.

The typical ECG in fascicular VT shows a RBBB morphology with a left anterior hemiblock pattern.^57^ There is typically a superior axis in the frontal plane, though it may be left superior with an RS in V5 and V6 or right superior with an RS or QS in these leads, depending on whether the origin is more proximal near the posteromedial papillary muscle (PM) or closer to the apical septum.^57^ QRS duration is typically narrow (< 140–150 ms) with a short RS interval, reflecting its origin from specialized conducting tissue **(Figure 6)**.^57^ Rarely, VT may involve the left anterior fascicle, resulting in a RBBB morphology and left posterior hemiblock pattern.^57,60^ More rarely, this may result in VT with a normal frontal plane axis, reflecting its origin in the upper LV septum.^57,60^

### Electrophysiology study and ablation

The retrograde transaortic approach is preferred for fascicular VT mapping and ablation, though the procedure can also be performed via transseptal puncture.^57^ With the ablation catheter in the LV, the septum is mapped from the apex to base to identify a target site. Once the site is identified, ablation can be performed with powers of 20 W to 40 W for one to two minutes.^57^ Typical effects on the arrhythmia include the progressive prolongation of the P1–P2 interval (see below), with slowing and subsequent termination of the tachycardia.^57^

**Figure 7:**
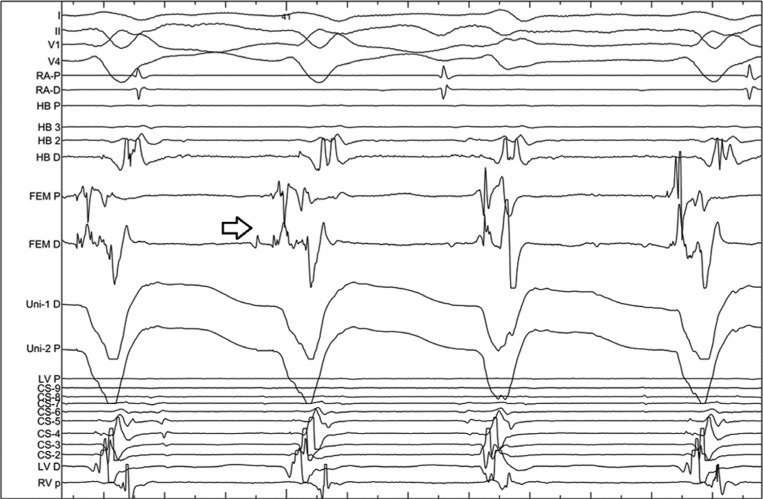
Mapping during fascicular VT. Note the EGMs recorded from the distal mapping catheter bipole. The first (P1) and second (P2) high-frequency potentials (arrow) precede His activation by 70 ms and 40 ms, respectively.

As mentioned above, fascicular VT is characteristically initiated by atrial extrastimuli and burst atrial pacing, though ventricular pacing is effective as well.^54,57^ In 90% to 95% of cases, the earliest ventricular activation is noted in the inferoposterior LV septum, corresponding with the location of the LPF.^57^ The local ventricular EGM is preceded by a high-frequency signal that is best recorded from the posterior one-third of the LV septum. This signal is felt to represent the activation of distal Purkinje tissue prior to exit from the LPF into the LV myocardium and is often referred to as P2 or the Purkinje potential (PP).^68^ A second discrete potential (often referred to as P1 or the pre-PP) is noted to precede P2 during VT and is felt to represent the entrance of the depolarizing wavefront into the abnormal Purkinje tissue (the verapamil-sensitive zone) in the proximal/mid-LV septum.^68^ Thus, the depolarizing wavefront during VT travels antegrade from the proximal/mid-LV septum through abnormal, slowly-conducting Purkinje tissue and retrogradely up the LPF, with exit into the myocardium in the inferoapical LV septum **(Figure 7)**.^57,69^

During sinus rhythm, antegrade activation occurs rapidly down the LPF, with simultaneous slow conduction through the abnormal Purkinje tissue. Because of the differences in conduction velocity in this region, the antegrade impulse is able to turn around and conduct retrogradely through the distal portion of the diseased Purkinje tissue.^57^ Thus, a catheter positioned near the LPF should be able to record a PP immediately preceding local ventricular activation and a delayed diastolic potential that represents retrograde activation of the slowly conducting limb of the circuit.^57^ A multipole catheter positioned in this region should be able to record both antegrade and retrograde diastolic potentials due to collision of the orthodromic and antidromic wavefronts.^57^ These late potentials should only be present in patients with fascicular VT, not in subjects with normally functioning Purkinje tissue.^68^ A similar phenomenon is seen during ventricular pacing from near the LPF, though the activation sequence should be reversed.^69^ In contrast, during VT, antegrade activation occurs slowly through the abnormal Purkinje tissue from the basal to the apical septum (hence, with an early P1 proximally on a multipole catheter) with rapid retrograde conduction through the LPF observed prior to exit into the LV myocardium.^57^ Entrainment of VT is typically performed from the RVOT, with an increase in the P1–P2 interval with a shorter pacing cycle length due to the decremental properties of the diseased Purkinje tissue.^57^

**Figure 8:**
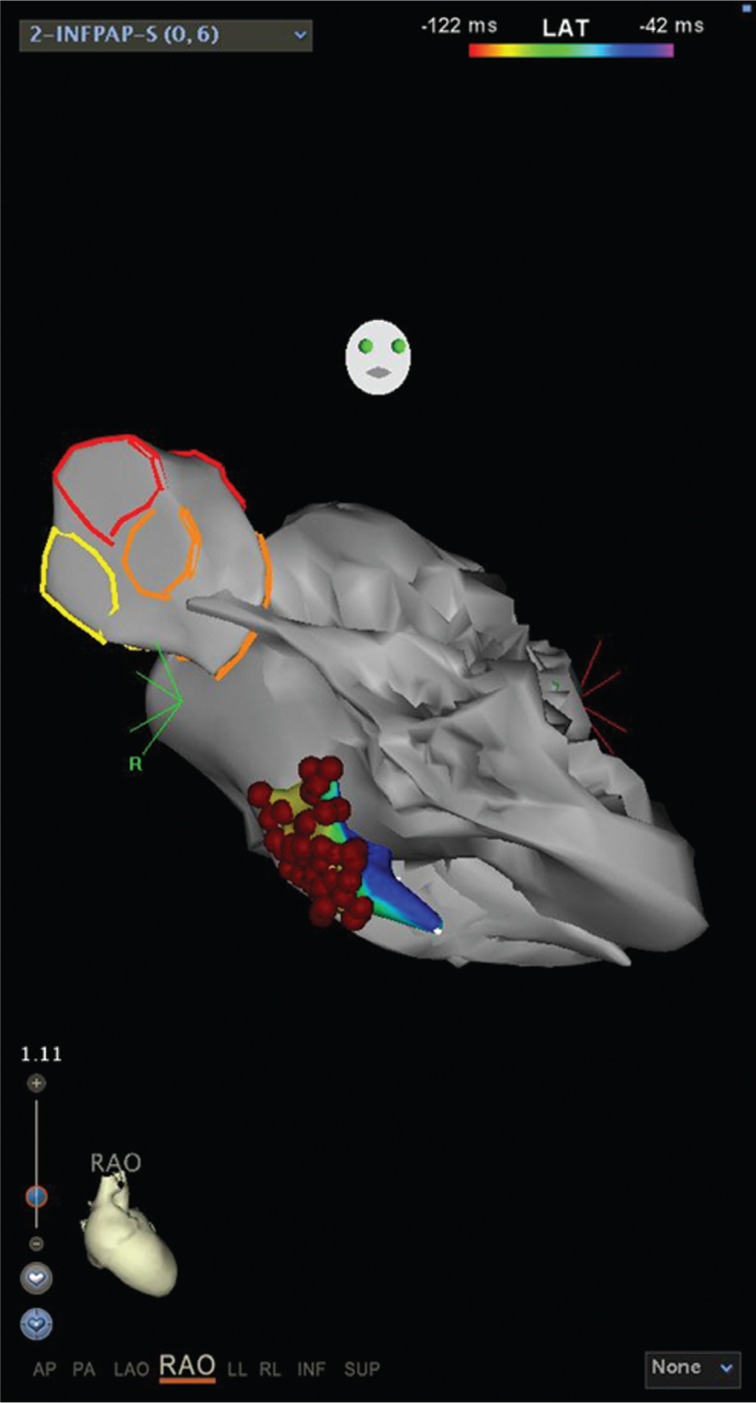
Right anterior oblique cranial view of the LV geometry, showing the successful ablation site for LPF VT. These images were created using CARTO^®^ 3 and the CARTOSound™ enhanced mapping feature (Biosense Webster, Diamond Bar, CA, USA).

Ablation is ideally performed during tachycardia to observe for slowing and termination and the importance of choosing the correct ablation site based on P1 and P2 timing.^57,70^ The optimal site of ablation is felt to be the earliest PP preceding QRS onset (typically 30–40 ms).^57^ This ablation corresponds to the turnaround point of the circuit in the posterior LV septum **(Figure 8)**.^57,70^ Notably, because the impulse has not yet exited into the LV myocardium at this location, ablation at the site of earliest ventricular activation will likely be ineffective, as P2 is typically located more basally.^57,70^ If pacing is performed at the earliest PP–QRS location, a stimulus–QRS time equal to the PP–QRS time predicts successful termination by ablation at that location.^71^ If there is uncertainty regarding a site with a particular PP–QRS interval, entrainment can be performed to confirm proximity to the tachycardia circuit.^57,70^ Ablation of fascicular VT has success and recurrence rates similar to those of ablation of OT tachycardias, at approximately 90% and 7% to 10%, respectively.^57,66^ In cases where VT is noninducible, one possible approach includes targeting the site of early P1 during sinus rhythm.^68,72^ Another option is ablating a line that transects the direction of conduction down the LPF during sinus rhythm.^72^

## Less common ectopic sites

### Papillary muscle ventricular arrhythmias

Recently, a group of patients without SHD were identified with VAs originating from the PMs of the LV.^73–79^ The prevalence of PM VAs has been reported as being 7% to 35% in patients with idiopathic VAs.^74,76,80^ Less is known about these arrhythmias than OT and fascicular VAs, though several common anatomic and clinical characteristics have been observed. Histologic studies have shown that the PMs have a rich network of Purkinje fibers and PM activation occurs at discrete connections between subendocardial Purkinje fibers at the base of the PMs with inhomogeneous electrical coupling.^81^ Interestingly, both patients with and without SHD who have PM VAs have been noted to have late gadolinium enhancement (LGE) on cardiac magnetic resonance imaging, though this clinical scenario remains poorly described in the literature and the relationship between LGE and arrhythmogenicity is unclear.^81^

Clinical characterization of patients with PM VT is limited by small study sample sizes. One study comparing patients with PM VT versus those with fascicular VT found PM VT patients to be significantly older (mean age: 77 ± 9 years versus 41 ± 7 years in the fascicular VT group), a trend mirrored in other small studies of PM VT patients.^74–79^ Sustained VT appears to be a less common presentation as compared with other idiopathic VAs, with more patients presenting with PVCs and nonsustained ventricular tachycardia.^1,2,4^ However, this finding is inconsistent across studies.^75,77,79^ The main symptoms reported across studies include palpitations and lightheadedness, with exertional dyspnea and syncope being less common.^73–79^

**Figure 9:**
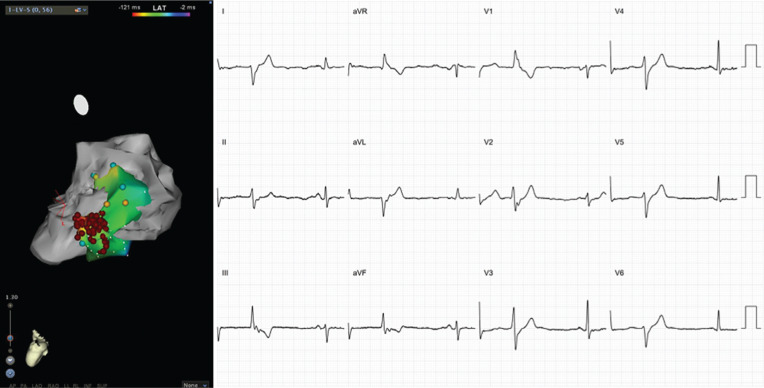
ECG and anatomic location of anterolateral PM PVCs. The left pane shows a posterolateral projection of the LV demonstrating successful ablation sites in the anterolateral PM. Note the atypical RBBB pattern and inferior axis on the surface ECG. These images were created using CARTO^®^ 3 and the CARTOSound™ enhanced mapping feature (Biosense Webster, Diamond Bar, CA, USA).

The ECG characteristics of PM VT are consistently reported as having RBBB morphology.^73,74,76,79^ However, in the above-referenced study comparing fascicular VT to PM VT, no patients with PM VT manifested the classic rSR’ pattern of RBBB.^74^ The frontal plane axis is suggestive of the specific PM origin, with posteromedial PM VT having a superior axis and anterolateral PM VT having an inferior axis **(Figure 9)**.^74,76^ As compared with fascicular VT, PM VT is reported to have a longer QRS duration and lower incidence of frontal plane Q-waves.^74^ A majority of patients in several studies of PM VT had QRS notching.^74,79^ However, it is unclear as to whether this information is useful for distinguishing PM VT from other idiopathic VAs or not. Across studies, PM VT more commonly arises from the posteromedial PM than the anterolateral PM.^82^

Invasive electrophysiologic studies of patients suspected of having PM VAs have provided clues regarding arrhythmia mechanism. Multiple studies report difficulty inducing ectopy with programmed stimulation and greater success with using intravenous isoproterenol or epinephrine.^73,74,77^ Few studies report on diagnostic maneuvers performed in these patients. However, one study noted that criteria for entrainment could not be demonstrated, suggesting that these VAs are automatic or triggered in nature. Earliest ventricular activation is frequently mapped to the PM base.^73,76,82^ This activation precedes surface QRS onset by approximately 20 ms to 40 ms.^73,74,76,79^ There is some discrepancy regarding the presence or absence of prepotentials preceding the local ventricular EGM. In a study by Good et al., 45% of patients with PM VAs had Purkinje potentials identified at the successful ablation site during both sinus rhythm and VA, though these occurred after QRS onset during sinus rhythm.^74^ In a larger series of patients, Yamada et al. noted no Purkinje potentials preceding EGM onset in sinus rhythm, but did observe the presence of a sharp prepotentials prior to the local ventricular EGM during VA.^76^ One study reported that the presence of a Purkinje potential preceding the local ventricular EGM was predictive of successful ablation.^77^ This finding possibly illustrates the importance of the previously mentioned Purkinje tissue present in the PMs in arrhythmogenesis. However, a majority of patients in one study^79^ who had high-frequency prepotentials during PM VAs did not have a Purkinje potential recorded during sinus rhythm, which the authors concluded may result from a different arrhythmogenic substrate within the PM itself. More research is required to determine the exact arrhythmogenic substrate and potential involvement of Purkinje tissue in arrhythmogenesis in these patients. Ablation in affected individuals is frequently difficult, owing to problems with catheter stability during ablation and adequate energy delivery. Greater long-term success is reported to be achieved with irrigated versus nonirrigated catheters, suggesting that the arrhythmogenic focus is located deeper within the muscle.^73,76,82^

### Mitral annular ventricular arrhythmias

A small subgroup of patients with idiopathic VAs have an arrhythmia origin around the mitral annulus (MA). The prevalence of MA VAs in patients with idiopathic VAs referred for ablation is reported to be 3% to 5%.^83–85^ Some studies have hypothesized that the majority of arrhythmias originate from the area of the left fibrous trigone or AMC.^84,85^ Few generalizations can be made about patients with these VAs given their relative rarity. However, similar to in the case of patients with PM VT, case series have suggested that patients with MA VAs are more likely to present with PVCs and nonsustained VT.^84–86^ These same patients are also less likely to present with syncope.^84–86^

**Figure 10:**
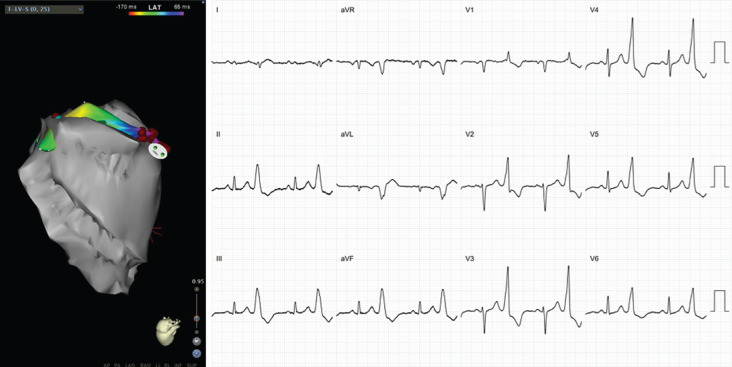
ECG and anatomic location of successful ablation sites for an AMC PVC. The left pane shows an LAO cranial view of a reconstructed LV geometry with ablation sites located in the great cardiac vein. Note the inferior axis and large R-waves throughout the precordium. These images were created using CARTO^®^ 3 and the CARTOSound™ enhanced mapping feature (Biosense Webster, Diamond Bar, CA, USA).

The ECG in patients with MA VAs shows RBBB morphology in lead V1 with monophasic R-waves or Rs complexes in leads V2 through V6 **(Figure 10)**.^84,85^ Arrhythmias arising from the anterior MA, left fibrous trigone, and AMC are more likely to have an inferior axis with monophasic R-waves in the frontal plane, while patients with VAs arising from the posterior annulus demonstrate some degree of S-wave in these leads.^85^ One study reported that VAs arising from the posteroseptal MA had a superior frontal plane axis and QS or rS complexes in leads I and aVL.^84^ As previously mentioned, a qR pattern in lead V1 is suggestive of an origin near the left fibrous trigone. A large series of patients with MA VAs was only able to identify longer QRS, intrinsicoid deflection, and pseudo-delta wave duration as being predictive of an epicardial origin.^85^ Similar to in PM VAs, QRS duration in MA VAs is significantly longer than in fascicular VAs, and MA VAs are more likely to have R ≥ S than fascicular and PM VAs are.^80^

Catheter ablation of MA VAs is safe and effective, with reported success rates ranging from 80% to 90%.^82,84^ Generally, the treatment of epicardial arrhythmias yields lower success rates.^82,84^ The electrophysiologic characteristics of these arrhythmias remain poorly described. Few studies have reported on diagnostic maneuvers aimed at elucidating details of arrhythmia mechanisms. However, multiple studies have reported inducibility with isoproterenol infusion, possibly supporting either a triggered or automatic mechanism.^84–86^ Some studies report high-frequency prepotentials during arrhythmia, though this outcome is inconsistent both across and within certain studies.^84,86^ A typical study frequently involves endocardial mapping using pacemapping and a comparison of the local ventricular EGM with QRS onset. If no good targets are found, mapping within the coronary sinus is performed, with the performance of coronary angiography used to assess the safety of ablation in this area.

## Idiopathic ventricular fibrillation

While the majority of VAs in patients with structurally normal hearts are benign, some patients present with polymorphic VT or with VF without any apparent SHD or identifiable inherited arrhythmia syndrome.^87^ The designation of these arrhythmias as “idiopathic” underscores the likely presence of an undetectable structural, genetic, metabolic, or other subclinical derangement. Many patients with inherited arrhythmia disorders were previously diagnosed with idiopathic VF prior to the identification of the causative gene (eg, Brugada syndrome, catecholaminergic polymorphic VT).^87^ Many structural cardiac abnormalities associated with SCD and VF are readily identifiable with modern imaging techniques. However, subtle structural abnormalities linked with infiltrative myocardial diseases (eg, cardiac sarcoidosis, arrhythmogenic right ventricular cardiomyopathy/dysplasia), or functional coronary abnormalities (eg, coronary artery spasm) may escape diagnosis in patients presenting with SCD. This lack of detection results in a diagnosis of idiopathic VF. Some authors regard ergonovine or acetylcholine testing for coronary artery spasm, endomyocardial biopsy, cardiac magnetic resonance imaging, and provocative testing for excluding Brugada syndrome (eg, ajmaline or procainamide challenge) as mandatory in patients presenting with otherwise idiopathic VF.^88^

Because of the rarity of idiopathic VF, it is difficult to draw any etiologic conclusions from the clinical and genetic associations with idiopathic VF that have been reported in the literature. Evidence for a monogenic origin of idiopathic VF comes from studies identifying causative mutations in genes encoding calmodulin.^89^ Other evidence also comes from studies of the ryanodine receptor^90^ and transcription factors related with the His-Purkinje system in families with idiopathic VF.^91^ However, even among patients with already identified inherited arrhythmia syndromes, a causative genetic mutation may not always be found.^92^ Separating true pathogenic from benign genetic polymorphisms may also be difficult.^92^ Further complicating this issue is the fact that several of the most common inherited arrhythmia syndromes have an ECG signature that may only intermittently be present (eg, Brugada syndrome). Thus, a patient who presents with idiopathic VF may have an underlying pathologic mutation or known arrhythmia syndrome, despite the presence of a normal surface ECG and negative genetic screening findings.^87^

Much work has previously been done to identify patients at risk of idiopathic VF based on surface ECG markers. For example, the early repolarization (ER) pattern observed in 1% to 5% of the general population was previously considered to be a benign finding.^93^ However, multiple studies have demonstrated that the ER pattern, particularly in the inferolateral leads of the surface ECG, is associated with an increased risk of idiopathic VF.^88,94^ This pattern is termed “early repolarization syndrome” when accompanied by SCD.^88^ Unfortunately, because of the high prevalence of an ER pattern in the general population and the relatively low prevalence of an ER pattern in patients with SCD, it is difficult to draw any direct mechanistic conclusions. Further, data regarding the ER pattern and idiopathic VF are somewhat unclear in the literature, as some authors identify ER syndrome and idiopathic VF as being two separate syndromes.^87,95^ Other evidence suggests that inheritance of the ER pattern is likely polygenic, with reports of mutations in *SCN5A*, *CACNA1C*, *CACNB2B*, and *KCNJ8* present in patients with VF and an ER pattern on ECG.^88^ Finally, while there is anecdotal evidence of familial inheritance of the ER pattern, inheritance of a malignant phenotype has not been reported.^88^

Because of these issues, patients with idiopathic VF are best understood as a heterogeneous group, including those with a likely unknown monogenic cause of VF, unrecognized structural or functional cardiac abnormalities, known but unapparent inherited arrhythmia syndrome, and a multifactorial cause of VF. Consequently, there is no single universally recommended testing strategy in patients presenting with idiopathic VF. Standard, targeted laboratory screening can be used to identify toxic and metabolic causes of arrhythmia. Further testing with cardiac structural imaging, ambulatory ECG monitoring, coronary angiography, provocative testing, and genetic testing are often dictated by the clinical situation, though the degree of testing required before a patient can truly be labeled as having idiopathic VF is a matter of debate.^87^ Unfortunately, only approximately 7% of patients who are initially diagnosed as having idiopathic VF receive a special diagnosis during follow-up.^96^ This number was historically near 30%, emphasizing the role of further research and clinical experience in arriving at a diagnosis in patients with idiopathic VF.^97^

Treatment of idiopathic VF consists almost exclusively of implantable cardioverter-defibrillator (ICD) therapy.^87^ Arrhythmia recurrence is high in patients with idiopathic VF, with almost one-third of patients experiencing a recurrence within five years.^98^ Predictors of recurrence ICD shocks have not been identified in idiopathic VF patients, though, as is true with other patients being treated via ICD therapy, the strongest predictor of inappropriate shocks is a history of atrial fibrillation (AF).^87^ In patients presenting with recurrent VAs or ICD shocks that are reliably precipitated by a short-coupled PVC, catheter ablation can be effective in reducing further recurrences.^87^

## Conclusions

VAs are common in patients without overt SHD. The most common underlying mechanisms are triggered activity from OT myocardium and reentry involving left-sided His-Purkinje tissue. The surface ECG is an essential first test to be performed in establishing the diagnosis and localizing the arrhythmia site of origin. Consequently, a detailed understanding of the anatomy and normal physiology of the cardiac conduction system and OTs is important in order to correlate electrocardiographic characteristics with recordings made during invasive electrophysiology study. These arrhythmias are readily amenable to catheter ablation and our understanding of their electrophysiologic behavior continues to evolve. The prognostic importance of VAs in these patients remains unclear, as most prior studies have not rigorously excluded SHD, except for clinical CAD. Still, patients who develop symptoms and/or LV dysfunction related with ventricular ectopy warrant therapy aimed at arrhythmia suppression.

A certain subset of patients without SHD present with polymorphic VT and VF. Such patients likely represent a heterogeneous group, composed of patients with known but not-yet-diagnosed inherited arrhythmia syndromes, subclinical SHD, or previously unidentified genetic variants that increase the risk of developing malignant VAs. Strategies aimed at identifying patients at increased risk are currently under investigation, though no single marker has emerged as clearly predictive. Even patients with a benign form of VA may present with polymorphic VT or VF, depending on the site or origin of the arrhythmia and its effects in terms of ventricular repolarization. As our understanding of the underlying genetic, anatomic, and cellular basis of these arrhythmias continues to improve, we will be able to further refine methods of diagnosis, risk prediction, and invasive and noninvasive management.
